# Urine MicroRNA as Potential Biomarkers of Autosomal Dominant Polycystic Kidney Disease Progression: Description of miRNA Profiles at Baseline

**DOI:** 10.1371/journal.pone.0086856

**Published:** 2014-01-29

**Authors:** Iddo Z. Ben-Dov, Ying-Cai Tan, Pavel Morozov, Patricia D. Wilson, Hanna Rennert, Jon D. Blumenfeld, Thomas Tuschl

**Affiliations:** 1 Laboratory of RNA Molecular Biology, Howard Hughes Medical Institute, The Rockefeller University, New York, New York, United States of America; 2 Molecular Pathology Laboratory, New York Presbyterian Hospital, Cornell University, New York, New York, United States of America; 3 Pathology and Laboratory Medicine, Weill Medical College, Cornell University, New York, New York, United States of America; 4 Centre for Nephrology, University College London Medical School, London, United Kingdom; 5 Rogosin Institute, Weill Medical College of Cornell University, New York, New York, United States of America; UCL Institute of Child Health, United Kingdom

## Abstract

**Background:**

Autosomal dominant polycystic kidney disease (ADPKD) is clinically heterogenic. Biomarkers are needed to predict prognosis and guide management. We aimed to profile microRNA (miRNA) in ADPKD to gain molecular insight and evaluate biomarker potential.

**Methods:**

Small-RNA libraries were generated from urine specimens of ADPKD patients (N = 20) and patients with chronic kidney disease of other etiologies (CKD, N = 20). In this report, we describe the miRNA profiles and baseline characteristics. For reference, we also examined the miRNA transcriptome in primary cultures of ADPKD cyst epithelia (N = 10), normal adult tubule (N = 8) and fetal tubule (N = 7) epithelia.

**Results:**

In primary cultures of ADPKD kidney cells, miRNA cistrons mir-143(2) (9.2-fold), let-7i(1) (2.3-fold) and mir-3619(1) (12.1-fold) were significantly elevated compared to normal tubule epithelia, whereas mir-1(4) members (19.7-fold), mir-133b(2) (21.1-fold) and mir-205(1) (3.0-fold) were downregulated (P<0.01). Expression of the dysregulated miRNA in fetal tubule epithelia resembled ADPKD better than normal adult cells, except let-7i, which was lower in fetal cells. In patient biofluid specimens, mir-143(2) members were 2.9-fold higher in urine cells from ADPKD compared to other CKD patients, while expression levels of mir-133b(2) (4.9-fold) and mir-1(4) (4.4-fold) were lower in ADPKD. We also noted increased abundance mir-223(1) (5.6-fold), mir-199a(3) (1.4-fold) and mir-199b(1) (1.8-fold) (P<0.01) in ADPKD urine cells. In ADPKD urine microvesicles, miR-1(2) (7.2-fold) and miR-133a(2) (11.8-fold) were less abundant compared to other CKD patients (P<0.01).

**Conclusions:**

We found that in ADPKD urine specimens, miRNA previously implicated as kidney tumor suppressors (miR-1 and miR-133), as well as miRNA of presumed inflammatory and fibroblast cell origin (miR-223/miR-199), are dysregulated when compared to other CKD patients. Concordant with findings in the primary tubule epithelial cell model, this suggests roles for dysregulated miRNA in ADPKD pathogenesis and potential use as biomarkers. We intend to assess prognostic potential of miRNA in a followup analysis.

## Introduction

Autosomal dominant polycystic kidney disease (ADPKD) is characterized by unpredictable progression rate and incidence of complications. Research of potential therapeutics is hampered by lack of short-term surrogate markers of therapeutic effects. Reduction in glomerular filtration rate is a late occurrence in the course of the disease that manifests only after >60% of normal renal parenchyma has sustained permanent damage.

The diagnostic criteria for ADPKD are based on renal ultrasonography and a positive family history [1]. Ideally, biomarkers of disease progression should reflect short-term changes in rate of cyst development, akin to blood pressure, cholesterol and glycated hemoglobin measurements to predict long-term benefits of respective medications. Accordingly, biomarkers could be used to support early detection, assess progression risk, monitor disease progression, identify factors involved in the disease pathogenesis, and inform on the success of therapeutic interventions [2]. Currently, biomarkers for ADPKD are lacking. Although there is an inverse association of GFR with total kidney volume, significant inter-subject variation in this relationship [3] limits its role as a biomarker for the individual patient. Recently, NMR spectroscopy of urine small molecules reliably discriminated ADPKD patients with moderately advanced disease from ADPKD patients with end-stage renal disease, patients with chronic kidney disease of other etiologies, and healthy siblings. The prognostic potential of these profiles was not studied [4].

MicroRNAs (miRNA) are small regulatory non-coding RNAs expressed in plants and animals. miRNA primary transcripts (pri-miRNA) are transcribed by RNA polymerase II from independent promoters, or processed from intronic regions, and are thus governed by regulatory mechanisms common to protein-coding genes. Accordingly, abundance of miRNAs, like protein coding mRNAs, is tuned both by cell-specific and cell non-specific regulation. Some miRNA are ubiquitous, while others are strictly limited to a cell type or lineage. Of approximately 600 known human miRNAs [5], most of which are detectable by deep-sequencing in a given cell type, only the highest expressed miRNAs are able to exert regulation on their target mRNA transcripts [6]. Developmental, physiologic and disease processes can alter miRNA levels. In addition to human cells, miRNAs are also found in body fluids. miRNA are released from cells, and are protected from extracellular nuclease activity by the miRNA ribonucleoprotein complex [7] or enclosing membrane vesicle [8]. Indeed, urine [9] has been used as source of protein biomarkers in polycystic kidney disease. Several features favor a role for miRNAs in biomarker discovery. Specifically, they can be assayed using high throughput platforms, are relatively simple to analyze compared to protein or mRNA profiling, and have been occasionally found to outperform mRNA-based biomarker discovery in cancer diagnosis [10], [11].

Our broad aim in this study is to examine miRNAs in ADPKD in an attempt to translate methodological advantages of miRNA profiling to the need for biomarkers in assessment of disease progression in ADPKD. miRNAs were profiled from nanogram amounts of input total RNA in clinical biofluid specimens from ADPKD patients and other chronic kidney disease (CKD) patients using deep sequencing of multiplexed small-RNA cDNA libraries. In the current report, we describe the urine miRNA profiles in relation to study group. In addition, since cystic proliferation of tubular epithelial cells is pivotal in ADPKD, we profiled miRNA in primary cultures of cyst-derived epithelial cells from ADPKD explants and normal kidneys as a cell culture model for the disease. We intend to evaluate prediction of kidney outcome by baseline miRNA abundance in a followup study.

## Subjects and Methods

### Ethics Statement

This study was approved by the Institutional Review Boards of the Rockefeller University Hospital and the Weill Medical College of Cornell University. Clinical data and specimens were collected after obtaining participants' written informed consent.

### Cell culture samples

Primary tubule epithelial cell cultures were derived from microdissected renal tubules and explanted kidney tissues from adults without parenchymal kidney disease prepared for transplant donation but deemed unsuitable due to renal vascular structural abnormalities (N = 8) or early stage ADPKD (N = 6, National Disease Research Interchange, Philadelphia); from surgical nephrectomy of end stage kidneys from patients with ADPKD (N = 4, PKD Foundation, Kansas) or from normal fetal kidneys after elective pregnancy termination (N = 7, Anatomic Gift Foundation, Philadelphia). Primary epithelial cells were grown to confluence in cell-type-specific supplemented media according to published techniques (**Figure S1**) [12]–[14]. Details of harvesting and expression markers in specimens used in this study were previously described [15]. RNA from immortalized tubular cells [16] and podocytes [17] were obtained for additional perspective.

### Urine specimens

We collected urine specimens from 20 patients with ADPKD and 20 patients with CKD of other etiologies, matched for age, sex, ethnicity/race and CKD stage (**Table 1**). Patients with active immune disorders affecting the kidneys were excluded. This component of the study is registered at *ClinicalTrials.gov* (identifier: NCT01114594). ADPKD patients enrolled in this study were also registered into an ongoing data repository (*ClinicalTrials.gov* identifier: NCT00792155). Total RNA was extracted from 50 ml urine sediment cells and from the ensuing cell-free, ultrafiltration-retained supernatant, a ∼250-fold concentrate of urine particles and macromolecules >100 kDa, thus including vesicle-enclosed RNA (VS2042, Vivaproducts Inc., Littleton MA) (see **Figure S2**). Small-RNA cDNA libraries were constructed and sequenced with modifications for small amounts of input RNA. Details of head-to-head comparison between precipitation-purified and silica column-purified RNA are shown in **Figure S3** [18].

**Table 1:** Clinical characteristics of study patients and biochemical features of study specimens*.

Type of specimen	N	Gender	Age, years	eGFR, ml/min/1.73 m^2^	CKD stage
		M/F	mean±SD [range]	mean±SD [range]	1/2/3/4
Primary cultures	ADPKD:10	n/a	n/a	n/a	n/a
	Normal: 8	n/a	n/a	n/a	n/a
	Fetal: 7	n/a	n/a	n/a	n/a
Urine specimens	ADPKD: 20	10/10	48±13 [25–70]	57±32 [20–130]	5/3/8/4
	CKD:20	10/10	50±12 [29–69]	57±26 [20–102]	5/3/8/4

* , Abbreviations: eGFR, estimated glomerular filtration rate; CKD, chronic kidney disease; ADPKD, autosomal dominant polycystic kidney disease; n/a, not assessed or not applicable.

### Sequencing and annotation of small-RNA cDNA libraries

Extracted total RNA was subjected to barcoded adapter ligation, and the reverse-transcribed small-RNA libraries were deep sequenced on Illumina Genome Analyzer II, as previously described [19]. The resulting sequence files were trimmed and split into the separate samples according to the barcode sequences. Extracted reads were assigned annotations by aligning to the genome and small-RNA databases. For miRNA annotation we used contemporary in-house definitions [5].

We display miRNA according to our recently published definitions [5]. Briefly, reads from multi-copy miRNA are summed into single entries. For example, miR-24-1 and miR-24-2 are represented by miR-24-1(2); the number in parenthesis symbolizes the number of members. Alternatively, reads from miRNA that share pri-miRNA cistronic transcription units are collapsed into miRNA cistron (precursor) clusters. For example, cluster-mir-23a(3) represents miR-23a, miR-24-2 and miR-27a. Lastly, miRNA with extensive sequence similarity are collapsed into sequence families. For example, miR-23a and miR-23b are members of seqfam-miR-23a(2). For analysis of differential expression we employ the cistron cluster arrangement, as cistron cluster members are co-transcribed and their levels are thus highly correlated [20]. However, when analyzing extracellular RNA (urine exosomes), secretion into the biofluid and stability in the biofluid affect miRNA levels, eliminating correlation between cistron cluster members [19].

### Statistical analysis

PASW 17.0 Statistics (www.spss.com) and the edgeR package for RNA sequencing on R platform [21], [22] were used for statistical analyses. Patient characteristics were compared between ADPKD and other-CKD groups using standard *t*-tests. Individual patient/sample miRNA data are displayed in supplementary tables as counts derived from deep sequencing reads. In addition, counts are aggregated according to miRNA sequence families and precursor cluster cistrons and presented in the respective supplementary tables (see above). Analyses of differential expression between ADPKD and non-ADPKD groups of samples were conducted under the assumption that gene (miRNA) counts in RNA deep sequencing data follow negative binomial distribution. The R/Bioconductor statistical package edgeR was developed with the negative binomial distribution assumption at its core. In generalized linear negative binomial models fitted with edgeR, the dependent variables are the genewise miRNA counts (expressed as counts per million miRNA counts, CPM) while the independent variable we included were disease group (e.g. ADPKD vs. other CKD urine cell pellets) and patient sex (when relevant). Fold-change values in miRNA abundance between the two groups of samples are calculated as the ratio of mean CPM in ADPKD to the mean CPM in the non-ADPKD group, and transformed to log base 2. P-values for coefficients in the model are computed by genewise likelihood ratio tests (or quasi-likelihood F-tests). Dispersion, which reflects biological variation, is estimated using the Cox-Reid method, and is inherent in the modeling procedure. Thus, within group variability impacts the statistical inference. Nominal P-values<0.05 are considered significant, provided that false detection rate (FDR) computed with the Benjamini-Hochberg method is less than 0.2.

## Results

We profiled miRNA in various specimens obtained from ADPKD and non-ADPKD patients. Clinical and biochemical characteristics of the study patients and samples are summarized in **Tables 1** **and** **2**, and further described in **Table S1 in File S1**.

**Table 2:** Clinical characteristics of study patients and biochemical features of study specimens*.

Type of specimen	Group	Total RNA	Total small RNA	Total miRNA	miRNA, fmol/µg	Urine miRNA
		ng/ml urine	read counts/10^6^	read counts/10^5^	of total RNA	fmol/l
Primary	ADPKD	n/a	1.0, 0.6–1.3	4.7, 2.7–7.7	4.2, 2.8–6.7	n/a
cultures	Normal	n/a	0.9, 0.9–1.1	5.1, 4.0–6.4	5.0, 3.2–9.5	n/a
	Fetal	n/a	1.3, 1.2–1.3	5.6, 4.3–6.2	6.9, 6.0–7.9	n/a
Urine cells	ADPKD	2.9, 1.4–11	3.5, 2.2–4.9	8.4, 4.4–16	8.1, 4.2–14	30, 8.2–83
	CKD	2.9, 1.4–7.8	3.5, 2.3–4.3	5.0, 2.7–13	7.4, 4.5–19	18, 5.6–163
Urine UF	ADPKD	0.3, 0.2–0.7**	0.48, 0.28–1.2	0.17, 0.12–0.26	6.4, 3.7–9.6†	1.8, 1.0–3.7
retentates	CKD	0.6, 0.3–0.8	0.55, 0.24–1.2	0.17, 0.07–0.35	2.8, 2.2–4.0	1.8, 0.9–4.0

* , Abbreviations: CKD, chronic kidney disease; ADPKD, autosomal dominant polycystic kidney disease; UF, ultrafiltration; n/a, not assessed or not applicable.

** , P-value = 0.056 (Mann-Whitney U test).

† , P-value = 0.02 (Mann-Whitney U test).

**Figure 1:**
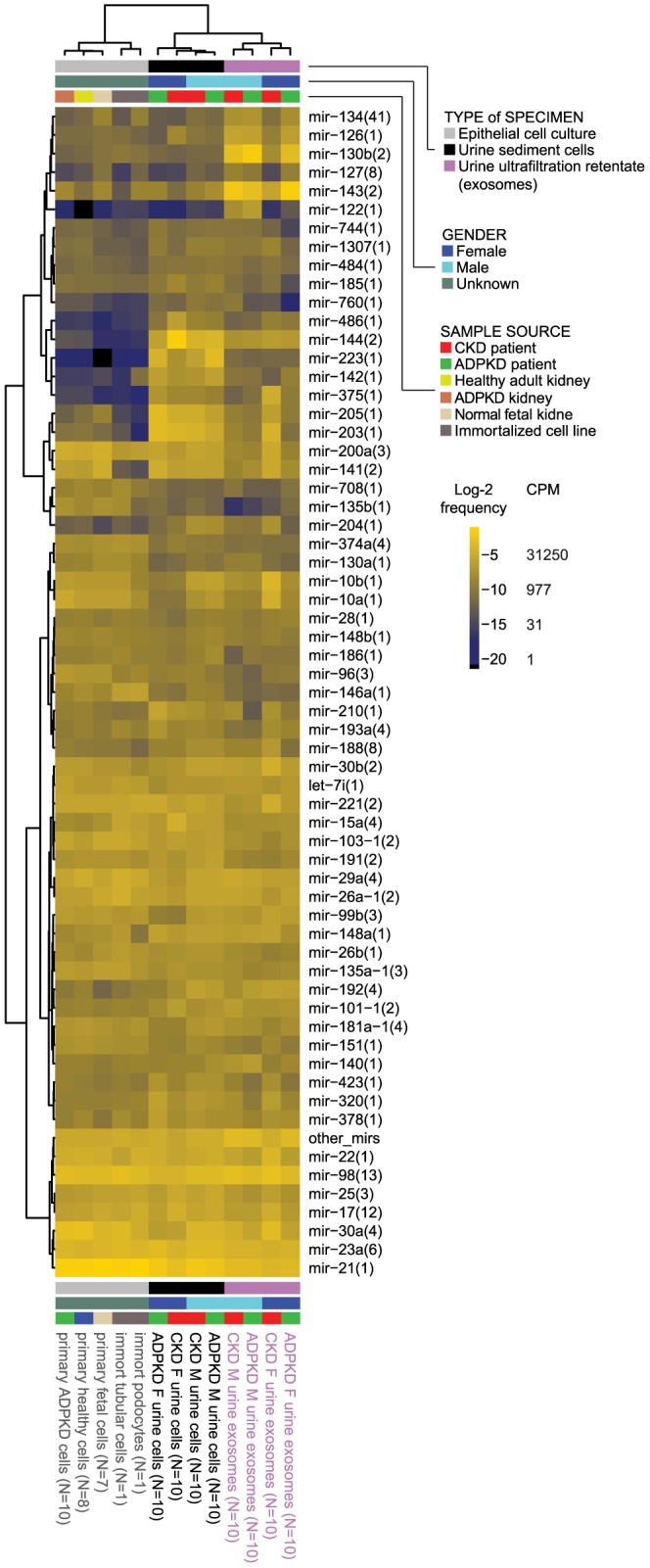
Hierarchically clustered study samples grouped by type and clinical characteristics. including additional non-study specimens for reference (columns) and miRNA cistrons (rows). High relative read frequency (log_2_-transformed) is indicated by bright yellow shades; low frequencies in dark blue. Corresponding numerical data is presented in the supplementary tables. Abbreviations: ADPKD, autosomal dominant polycystic kidney disease; CKD, chronic kidney disease; immort, immortalized; CPM, counts per million; F, female; M, male.

**Figure 1** provides an outline perspective of the abundant miRNA across the study samples, aggregated by gender and specimen type, and presented as a heatmap. In the following sections, findings are depicted according to the type of specimen: primary cultures of human kidney cyst-lining or normal tubular epithelia as *ex vivo* disease model; urine sediment cells; and microvesicles retained following urine centrifugation and ultrafiltration.

### MicroRNA content in primary cultures of renal tubule epithelia

Small-RNA categories were obtained from primary tubule epithelial cell cultures grown from microdissected renal tubules of adults (i) without parenchymal kidney disease (N = 8), (ii) with early stage (pre-dialysis) ADPKD (N = 6), (iii) end-stage ADPKD kidneys (N = 4), and from normal fetal kidneys (N = 7) (**Table S1 in File S1**). Based on the addition of defined amounts of synthetic calibrator oligoribonucleotides [23], the miRNA content in input total RNA was 5.8 fmol/µg (interquartile range (IQR) 3.5–7.9 fmol/µg), with no significant differences between study groups.

miRNA profiles diverged in accordance with sample groups (**Figure S4**). Sequence reads derived from let-7i(1) (2.3-fold), mir-143(2) (9.1-fold) and mir-3619(1) (11.8-fold) were upregulated, and mir-1(4) (20-fold), mir-133b(2) (20.8-fold) and mir-205(1) (3.1-fold) cistron members were downregulated in primary cultures of cyst epithelial cells compared to normal kidney-derived cells (P-values<0.01, FDR<0.2) (**Table 3**) (see **Tables S2, S3, S4, S5 in File S1** for complete count data and miRNA cluster and sequence family definitions). Resembling ADPKD-derived cells, fetal cells had similarly lower levels of mir-1(4) and mir-133b(2) members and higher levels of mir-143(2) and mir-3619(1) members compared to normal adult tubular epithelial cells (P-values<0.05, FDR<0.2) (**Table 3**).

**Table 3:** miRNA cistron clusters differentially expressed between ADPKD-derived and normal kidney-derived primary tubular epithelial cultures*.

		ADPKD (N = 10) vs. normal adult (N = 8)			fetal (N = 7) vs. normal adult (N = 8)		
	CPM (log_10_)	FC (log_2_)	P-value	FDR	FC (log_2_)	P-value	FDR
cluster-hsa-let-7i(1)	4.1	1.2	3.7E-04	2.6E-02	−0.1	7.6E-01	8.9E-01
cluster-hsa-mir-143(2)	3.5	3.2	3.6E-03	1.5E-01	2.3	2.8E-02	1.2E-01
cluster-hsa-mir-205(1)	2.9	−1.6	9.1E-05	8.9E-03	1.9	5.0E-05	9.8E-04
cluster-hsa-mir-1-1(4)	2.8	−4.3	4.5E-04	2.6E-02	−5.8	2.0E-04	3.1E-03
cluster-hsa-mir-133b(2)	1.2	−4.4	2.9E-05	8.6E-03	−4.5	7.6E-05	1.4E-03
cluster-hsa-mir-3619(1)	0.8	3.6	8.1E-05	8.9E-03	2.0	3.6E-02	1.4E-01

* , Cutoff for presentation in this table is p-value<0.05 and FDR<0.2 in the ADPKD vs. normal comparison.

Segmental origin of the cultured cells, determined in all specimens except end-stage ADPKD kidneys, e.g. proximal straight tubule (PST), thick ascending limb (TAL) and collecting duct (CT) had no impact upon miRNA profiles. However, cells originating in cysts of end-stage ADPKD kidneys had higher content of mir-21(1) (1.6-fold), mir-30a(4) (1.5-fold) and mir-101(2) (3.2-fold) and lower content of mir-143(2) (10-fold), compared to PST, TAL or CT-derived cells (P-values<0.01, FDR<0.2). For perspective, we provide miRNA profiles of immortalized kidney epithelial cell lines of tubular and podocyte origin (**Figure 1** and see **Tables S6, S7, S8 in File S1** for details and differential expression). None of the top 15 miRNA precursor clusters or top 40 miRNA sequence families were differentially expressed in immortalized compared to primary cells (**Table S8 in File S1**), suggesting that miRNA expression and regulatory function in immortalized cell lines may recapitulate their respective properties in primary cultures of kidney epithelial cells. Presence of SV40-miR-S1 and downregulation of miR-141/miR-200c in immortalized cells were the most striking differences in miRNA profiles compared to primary cells (**Table S7 in File S1**).

### MicroRNA content in urine of ADPKD and other CKD patients

We collected urine specimens from 20 patients with ADPKD and 20 patients with chronic kidney disease (CKD) of other etiologies, matched for age, sex, ethnicity/race and CKD stage (**Table 1**). Yields of total RNA extracted from urine sediment cells and from cell-free, ultrafiltration-retained supernatant specimens (containing microvesicles) and small-RNA annotation categories are summarized in **Table 2** **and Table S1 in File S1**. In *sediment cell* RNA, total RNA yield (2.9 ng per ml of urine), small-RNA annotation categories and calculated total miRNA content (7.4–8.1 fmol per µg total RNA) did not differ by disease group or gender. In *cell-free ultrafiltration-retained material* total RNA yields were 2-fold lower, but miRNA content per µg total RNA were 2.3-fold higher in ADPKD compared to non-ADPKD CKD specimens, and thus molar miRNA concentrations were overall similar; 68–104 fM (**Table 2**, and see also **Table S1 in File S1**).

Abundance of specific miRNA in urine cells and cell-free ultrafiltration-retained specimens is depicted in **Tables S9, S10, S11, S12, S23, S14 in File S1**. Compared with CKD of other etiologies, in urine cells from ADPKD mir-143(2) cluster members were upregulated, whereas mir-1(4) and related mir-133b(2) were downregulated. Moreover, there was an increased relative abundance of mir-223(1), mir-199a(3) and mir-199b(1) in ADPKD (**Table 4**). In cell-free urine ultrafiltration-retained specimens, relative abundance of mir-1(4) cistron members was lower in ADPKD compared to other CKD patients (**Table 5**).

**Table 4:** miRNA cistron clusters differentially expressed between ADPKD (N = 20) and other CKD (N = 20) patient urine sediment cells*.

	log_10_CPM	log_2_FC	P-value	FDR
cluster-hsa-mir-223(1)	4.6	2.5	1.3E-06	1.7E-04
cluster-hsa-mir-142(1)	3.9	1.3	3.8E-03	5.8E-02
cluster-hsa-mir-143(2)	3.4	1.6	1.6E-04	6.9E-03
cluster-hsa-mir-133b(2)	3.3	−2.3	3.2E-03	5.2E-02
cluster-hsa-mir-652(1)	3.1	0.9	6.3E-03	9.1E-02
cluster-hsa-mir-338(1)	2.6	1.9	2.7E-05	2.2E-03
cluster-hsa-mir-450a-1(4)	2.5	1.2	4.4E-04	1.1E-02
cluster-hsa-mir-199a-1(3)	2.5	1.4	1.2E-03	2.5E-02
cluster-hsa-mir-199b(1)	2.4	1.8	7.8E-05	4.8E-03
cluster-hsa-mir-582(1)	2.4	1.6	2.7E-04	8.3E-03
cluster-hsa-mir-3613(1)	2.1	1.0	1.0E-02	1.4E-01
cluster-hsa-mir-1-1(4)	2.0	−2.1	3.6E-04	9.8E-03
cluster-hsa-mir-618(1)	1.8	1.5	2.7E-03	4.8E-02
cluster-hsa-mir-2115(1)	1.6	1.4	2.3E-04	7.9E-03
cluster-hsa-mir-873(2)	1.5	1.2	1.7E-03	3.2E-02
cluster-hsa-mir-2355(1)	1.5	1.9	1.4E-06	1.7E-04
cluster-hsa-mir-551a(1)	1.2	1.8	1.7E-04	6.9E-03
cluster-hsa-mir-139(1)	1.1	−1.4	1.1E-03	2.4E-02
cluster-hsa-mir-370(1)	0.8	−1.9	1.5E-02	2.0E-01

* , Cutoff for presentation in this table is p-value<0.05 and FDR<0.2.

**Table 5:** Mature miRNA differentially expressed between ADPKD (N = 20) and other CKD (N = 20) patient urine exosomal preparations*.

	log_10_CPM	log_2_FC	P-value	FDR
hsa-miR-133a(2)	4.3	−4.0	5.0E-04	3.9E-02
hsa-miR-1(2)	4.1	−3.1	6.8E-04	3.9E-02
hsa-miR-671	4.0	1.9	6.3E-03	2.4E-01
hsa-miR-378	4.0	−1.6	1.4E-02	3.9E-01
hsa-miR-221	3.8	1.1	3.5E-02	7.9E-01
hsa-miR-98	2.7	−3.4	4.7E-02	8.7E-01

* , Cutoff for presentation in this table is p-value<0.05.

### MicroRNA sequence variants in primary cultures of normal, cyst-lining and fetal cells

**Figure 2:**
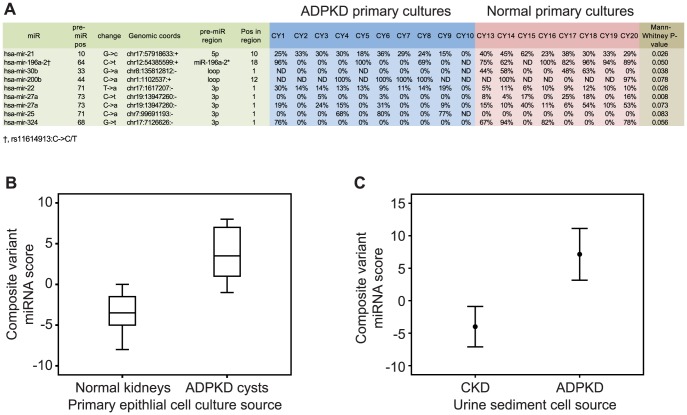
miRNA sequence variants that were found to be differentially expressed between ADPKD and non-ADPKD specimens. (**A**) miRNA sequence variants that were found to be differentially expressed between ADPKD cyst-derived primary cultures and normal adult kidney derived cultures (see complete list in Table S15 in File S1). (**B**) Box plot showing a composite score derived from the information in (A). The median frequency of each miRNA variant across the samples was used to generate the score; 1 point was added (or reduced) for each miRNA variant with frequency above (or below) the median frequency. (**C**) Bar plot showing a composite score derived in a manner similar to (B) from patient' urine sediment cell sequence data (contributing miRNA variants are depicted in Table S16 in File S1).

We found 138 unique miRNA sequence variants across primary culture specimens relative to the reference genome, according to published criteria (**Table S15 in File S1**) [5]. Sequence variants are predominantly due to RNA editing but may represent genetic variation. Non-parametric analysis revealed that the relative abundance of 9 sequence variants (related to 8 unique miRNAs) was altered in primary ADPKD cells compared to normal cells (at α = 0.1, **Figure 2A**). A composite score derived from the relative expression of these sequence variants (the score was defined as the number of variants with expression above median minus number of variants with expression below median) strongly discriminated ADPKD from normal renal cells (**Figure 2B**; area under the ROC curve 0.975, P = 0.001). Intriguingly, relative expression of sequence variants in urine sediment cell RNA (**Table S16 in File S1**) followed similar trends for 6 of the 8 miRNA above, and a similarly derived score discriminated ADPKD from other CKD patients' urine cells (**Figure 2C**; area under the ROC curve 0.713, P = 0.037).

### Mining of small-RNA reads to discover novel microRNA

Examination of sequence reads (21 to 23 nt length) that mapped to the human genome but had no matching small-RNA annotation in primary culture libraries (normal-, ADPKD- and fetal-derived cells) revealed that no single sequence exceeded a level corresponding to 0.02% of total miRNA (i.e., 200 reads per million miRNA reads). A miRNA of this level typically ranks ∼250 in abundance (namely, be outcompeted in terms of argonaute occupancy and potential for target repression by ∼250 higher-ranked miRNA). Thus, the existence of a previously unidentified yet biologically important kidney tubule epithelial miRNA is effectively ruled-out [6].

## Discussion

In this study, we obtained expression profiles of miRNA in ADPKD patient-derived primary cell cultures and urine RNA. Sequence profiles were generated by sequencing of multiplexed small-RNA cDNA libraries constructed in-house from as low as 5 ng total RNA per sample. This technical achievement has not been reported with currently available commercial kits [24], [25]. Advantages of multiplexing include shorter processing time, less labor-intensive, lower sequencing costs (we estimate 25 USD per sample for reagents and sequencing at 5 Mio reads/sample plus 75 USD per sample for labor), minimization of batch effects and thus improving the prospects for clinical research utility [23].

We studied primary cultures of epithelial cells lining ADPKD cysts as a basic model of the disease [12], [14], [26]. Similar cell systems were also reported to harbor phenotypic differences compared to normal cells, such as higher rates of proliferation, secretion and cell-matrix adhesion, decreased cell migration and abnormal polarization of specific membrane proteins such as EGFR [27]–[29]. Examination of miRNA profiles generated from these cells showed modest differences between ADPKD and normal renal epithelial cells. These differences were to an extent reminiscent of changes seen between normal fetal and adult cells. In line with the previously reported failure to switch-off fetal proteins [30], this finding may point to transcriptional alterations that contribute to the pathogenesis of cyst formation.

Compared to normal cells, ADPKD and fetal cells had lower levels of mir-1(4) and mir-133b(2) and higher levels of mir-143(2) and mir-3619(1) members. mir-143(2) members (predominantly miR-145) are highly abundant throughout the urinary tract, particularly in the lower segments. In contrast, mir-1(4) and mir-133b(2) are not abundantly expressed in kidneys (see below). Nonetheless, mir-1(4) and mir-133b(2) were reportedly downregulated in renal cell carcinoma (RCC) specimens and cell lines, and transfection experiments suggested that these clusters of miRNAs may be tumor suppressive in the kidney [31]. While epidemiological data do not demonstrate that patients with ADPKD bear an increased risk for RCC [32], a recent surgical specimen-based study showed 5% prevalence of RCC in ADPKD [33]. It is thus intriguing that in cultures of both fetal tubule cells and in cells obtained from ADPKD cysts, levels of mir-1(4) and mir-133b(2) cluster members were consistently lower (∼25–60-fold) than in normal kidney cells, in which they constituted ∼0.19% of all miRNA (ranking 41 of 350 detectable miRNA clusters). It should be noted that mir-1(4) and mir-133b(2) have crucial roles in muscle development. Transcription of mir-1(4) clusters is regulated by prototypical myocyte differentiation factors [34] and mir-1(4) members inhibit cardiomyocyte growth and proliferation [35]. In our miRNA expression database, respective abundance of mir-1(4) and mir-133b(2) was 27.2% and 0.18% in fetal heart; 19.4% and 0.14% in adult heart; and 54.1% and 2.3% in adult skeletal muscle (Williams Z and Tuschl T, unpublished). In light of these abundance data, the functional importance of mir-1(4) and mir-133b(2) members in kidney cells, in which their level of expression is ∼100-fold lower than myocytes, remains to be clarified.

We hypothesized that transcriptome-wide urine miRNA profiles would differ between ADPKD and other chronic kidney diseases, and that alterations discovered in the primary culture model may be recapitulated in the patient specimens. Indeed, abundance of specific miRNA in cellular and extracellular (exosome) specimens differed in ADPKD and non-ADPKD patients. Increased levels of mir-143(2) (urine cells) and decrease in mir-1(4) and mir-133b(2) members (urine cells and extracellular RNA) were found in ADPKD patients, extending the findings from primary cultures to clinical specimens. mir-499(1), albeit rare in kidney cells, was also significantly lower in ADPKD cell-free urine RNA compared to non-ADPKD specimens. miR-499 is encoded within an intron of the sarcomeric myosin gene, Myh7b [36], providing an additional link between ADPKD and suppression of muscle-enriched miRNA.

We observed higher levels of the lymphocyte/monocyte associated miR-223 and fibroblast-enriched miR-199a and miR-199b in ADPKD urine specimens. Monocyte infiltration [37]–[39] and myofibroblast transition [40] have been implicated in the pathogenesis of ADPKD. Moreover, increased levels of urine monocyte chemoattractant protein-1 (MCP1), possibly secreted by cyst epithelium, has been shown to precede increases in serum creatinine in ADPKD [41] is a potential biomarker [42]. This implies that participation of these cell types in the pathogenic process can be detected and monitored by examining spot urine samples.

In addition to analyzing differential expression (also possible with RT-PCR and hybridization-based profiling methods), we made use of information uniquely attainable by RNA sequencing. In primary culture profiles, we searched for yet undiscovered human miRNA. However, since miRNA action depends on stoichiometric interaction with their mRNA targets, exceedingly rare miRNA, even if processed and handled as prototypical miRNA, are unlikely to convey detectable post-transcriptional regulation. A study employing large-scale miRNA decoy and sensing libraries combined with RNA sequencing showed that only the most abundant miRNAs in a cell mediate target suppression and that ∼60% of detected miRNAs had no discernible activity [6]. The authors proposed that miRNAs expressed at levels below 1,000 reads per million (RPM, namely 0.1% of all miRNA) do not mediate substantial regulation on natural targets. Accordingly, our finding that no un-annotated sequence in the cultured cell libraries surpassed 200 RPM practically rules-out the existence of unidentified miRNA likely to mediate target suppression in kidney tubule epithelium. Conversely, similar considerations *support* a functional role for mir-1(4) and mir-133b(2) members in normal kidney epithelial cells, in which they are present at ∼1,900 RPM (0.19%), see above.

Genome-encoded and post-transcriptional alterations in miRNA sequence have bearings on miRNA stability and target repression [43], and may contribute to the biomarker potential of miRNA profiling. We mined our small-RNA libraries for variation in miRNA sequence and found evidence for previously reported miRNA SNPs and both reported and unreported RNA modifications. A subgroup of these modifications was associated with disease etiology in both primary cultures and urine sediment cells. These findings represent another unique advantage of RNA sequencing in the process of biomarker discovery.

## Conclusion

We profiled miRNA in ADPKD and non-ADPKD-derived kidney epithelial cells and in two sets of ADPKD and non-ADPKD patient urine specimens. We propose a role for repression of mir-1(4) and mir-133b(2) members in the pathogenesis of ADPKD, and for their potential use as biomarkers for monitoring disease progression and response to evolving treatments. However, prospective, longitudinal studies of these miRNA members are required to establish their role as biomarkers of ADPKD. Indeed, we intend to examine prediction of ADPKD progression in a followup longitudinal study of the study participants reported herein. We also suggest that enrichment of monocyte- and fibroblast-specific miRNA in urine from ADPKD patients' urine supports their role in disease progression. Finally, we have demonstrated the feasibility of multiplexed small-RNA cDNA library preparation and sequencing from nanogram-scale input total RNA, and its utility in generating research hypotheses and in the process of biomarker discovery.

## Supporting Information

Figure S1 **Phase contrast images of confluent normal renal tubule and ADPKD cystic epithelial cell monolayers.** (**A**) primary fetal, (**B**) primary adult, (**C**) primary early ADPKD and (**D**) primary end-stage ADPKD.
(EPS)

Figure S2 **Electron micrographs of a representative urine ultrafiltration retentate specimen showing vesicular structures of different sizes**; uranyl-acetate (‘negative’) stain.
(EPS)

Figure S3 **Parallel miRNA profiling from selected split urine ultrafiltration retentate samples.** (exosome preparations). (**A**) Experimental design – urine specimens were homogenized with TRIzol LS (Life Technologies – Invitrogen), RNA was extracted with chloroform, and the aqueous phase split and subjected to either isopropanol precipitation or miRNeasy silica column purification (Qiagen). For precipitation, glycogen (15 µg) was added prior to isopropanol, and precipitation carried out overnight at −20°C. (**B**) Differentially extracted miRNA (silica column vs. isopropanol precipitation) as determined with edgeR [22] by adjusting for specimen (blocking factor). Seven miRNA were differentially extracted at P-value<0.05; however, false detection rate (FDR) was >0.2 in all cases. Six of seven miRNA were enriched in column purifications compared to precipitation. Average GC content of enriched miRNA was 51% (range 36 to 68%), and average minimum free energy of optimal secondary structure −2.0 kcal/mol (range −3.5 to 0 kcal/mol). As opposed to Kim YK et al [18], we did not observe depletion of miR-141 or other miRNA with low GC content and stable secondary structure. We suggest that prolonged incubation in the presence of glycogen as co-precipitant may account for relatively more efficient recovery of these miRNA by isopropanol precipitation in our study [18].
(EPS)

Figure S4 **miRNA profiles in primary cultures of renal tubule epithelia.** (**a**) Hierarchically clustered samples (columns) and miRNA cistrons (rows). High relative read frequency (log2-transformed) is indicated by bright yellow shades; low frequencies in dark blue. Selected miRNA cistrons are color-shaded to denote dysregulation in ADPKD-descended cultures. (**b**) MA plot generated showing miRNA cistron differential expression between normal adult kidney and ADPKD cyst-derived epithelial cultures (R/Bioconductor; ‘edgeR’ package). (**c**) Multidimensional scaling (MDS) plot of primary culture samples according to miRNA cistron expression profiles (R/Bioconductor; ‘limma’ package).
(EPS)

File S1 Contains. **Table S1. Small RNA annotation categories across types of study samples, given as percentage of all post-filtering reads. Table S2. Tuschl lab miRNA group definitions. Table S3. Merged mature miRNA profiles of primary kidney cell cultures. Table S4. miRNA precursor (cistron) cluster profiles of primary kidney cell cultures. Table S5. miRNA sequence family profiles of primary kidney cell cultures. Table S6. Merged mature miRNA profiles of pooled primary and immortalized kidney cell cultures. Table S7. miRNA precursor (cistron) profiles of pooled primary and immortalized kidney cell cultures. Table S8. miRNA sequence family profiles of pooled primary and immortalized kidney cell cultures. Table S9. Merged mature miRNA profiles of patients' urine sediment cells. Table S10. iRNA precursor (cistron) cluster profiles of patients' urine sediment cells. Table S11. miRNA sequence family profiles of patients' urine sediment cells. Table S12. merged mature miRNA profiles of patients' urine exosome preparations. Table S13. miRNA precursor (cistron) cluster profiles of patients' urine exosome preparations. Table S14. miRNA sequence family profiles of patients' urine exosome preparations. Table S15. miRNA sequence variants in profiles generated from primary kidney cell cultures. Table S16. miRNA sequence variants in profiles generated from patients' urine sediment cells.**
(XLS)
